# COVID-19 and the uniqueness of South Africa

**DOI:** 10.4102/sajid.v35i1.208

**Published:** 2020-06-15

**Authors:** Jarrod Zamparini, Jacqui Venturas, Nina Diana

**Affiliations:** 1 Division of Infectious Diseases, Department of Medicine, Charlotte Maxeke Johannesburg Academic Hospital, Johannesburg, South Africa; 2 Department of Internal Medicine, Faculty of Health Sciences, University of the Witwatersrand, Johannesburg, South Africa; 3 Division of Nephrology, Department of Medicine, Charlotte Maxeke Johannesburg Academic Hospital, Johannesburg, South Africa

Severe acute respiratory syndrome coronavirus 2 (SARS-CoV-2), a bat-borne betacoronavirus, was first identified in December 2019 from bronchoalveolar lavage specimens obtained from five patients in Wuhan, China who presented with severe community-acquired pneumonia of unknown aetiology.^1^ In February 2020, the disease caused by this virus was designated COVID-19. The number of cases spread rapidly throughout the world and was labelled a pandemic by the World Health Organization (WHO) on 11 March 2020, with the first case in South Africa reported on 5 March 2020 in a traveller returning from Italy. To date there have been 10,015 cases in South Africa and 194 deaths.^2^

The presentation of COVID-19 resembles a mild flu-like illness in the majority of patients (81%). Severe disease is seen in 14% and critical disease (including acute respiratory distress syndrome [ARDS], multiorgan dysfunction and shock) in 5%.^3^ Presentation in the pneumonic stage of COVID-19 is clinically similar to other causes of severe community-acquired pneumonia. In the South African population *Streptococcus pneumoniae*, influenza virus, *Mycobacterium tuberculosis* and *Pneumocystis jirovecii* should also be considered.^4^ Importantly, co-infection with other respiratory viruses has been reported to be up to 22% in an American case series,^5^ with reports from China indicating 50% co-infection with other respiratory bacteria and viruses.^6^ In Wuhan, 87% of cases occurred in the 30 to 79 year age group, with an overall case fatality rate (CFR) of 2.3%. The CFR was significantly higher in patients ≥ 80 years old (14.8%) and in those with co-morbid disease, particularly cardiovascular disease (10.5%), diabetes (7.3%) and hypertension (6%).^3^

Of concern, the South African adult population has high rates of hypertension (26.9%) and diabetes (11.3%).^7^ In addition to this, it is estimated that there are 7.97 million people in South Africa living with HIV^8^ (of which only 55% are virally suppressed).^9^ It is unclear what the effect of co-infection with HIV has on patients infected with SARS-CoV-2, and whether an anti-retroviral regimen that includes lopinavir/ritonavir may have a protective effect. A study from China reported similar rates of COVID-19 disease in HIV positive and HIV negative patients.^10^ In this cohort, disease outcome was related to age and other co-morbid disease, rather than CD4 count, HIV viral load and the anti-retroviral therapy.

Currently there is no specific treatment for COVID-19. Most guidelines recommend supportive therapy with oxygen, empiric broad-spectrum antibiotics and antipyretics. Specific agents tried include remdesivir, chloroquine, azithromycin, lopinavir/ritonavir, interferon, intravenous immunoglobulin, corticosteroids, IL-6 inhibitors (Tocilizumab) and IL-1 inhibitors (Anakinra). Evidence is based on in-vitro and observational studies. The only randomised controlled trial using lopinavir and ritonavir (approved for the treatment of HIV infection) showed no difference in the time to resolution of symptoms.^2^

A large number of trials for a specific SARS-CoV-2 vaccine are ongoing.^11^ In the meantime interest has developed surrounding the possibility that the tuberculosis vaccine, BCG (Bacille Calmette Guerin) may induce ‘trained immunity’ by epigenetic remodelling, which would alter immune responsiveness to viral infections. It has been suggested that COVID-19 seems to be worse in countries with an absent or more recent BCG vaccination policy.^12^ A prospective trial is under way in Australia to determine whether BCG vaccination reduces the incidence and severity of COVID-19.^13^ BCG has been part of the immunisation schedule in South Africa since 1973; this may offer an element of protection.^14^

In South Africa, COVID-19 also raises socioeconomic concerns. Approximately 5.5 million people live in densely populated informal settlements where ‘social distancing’ is difficult, if not impossible. Overall only 74.2% of South Africans have access to water in their houses or on their properties; this number is even lower (40.4%) in rural areas, which means that regular handwashing may be unrealistic in these communities.^8^ In addition, the ratio of doctors and nurses per person is far lower than many European countries that have been hardest hit by the COVID-19 outbreak. In 2017 in South Africa there was one doctor per 1111 people and one nurse per 284 people, 4.5 and 1.6 times less than Italy, which has one of the highest case fatality rates to date.^15^

COVID-19 has spread rapidly across the globe, affecting nearly every country and plunging the world into a state of pandemic not seen in more than a century. It has invaded every facet of our connected lives and is foremost in the mind of every clinician and citizen. Despite this, we must be cognisant of the need not to neglect other diseases and comorbidities during this time. We have the advantage of being able to see how the pandemic has unfolded in the rest of the world, allowing us to learn from their clinical and political response. The South African government enacted an early lockdown, in the hope of flattening the curve and avoiding a collapse of its already stretched healthcare services. Lessons learnt in terms of management, both clinical and logistic, have allowed us to prepare as best as we can.
